# Duodenal Obstruction and Grade III Acute Cholangitis Following Pancreaticoduodenal Artery Aneurysm Rupture During Conservative Management of Acute Type B Aortic Dissection: A Case Report

**DOI:** 10.7759/cureus.115546

**Published:** 2026-08-31

**Authors:** Osamu Kinoshita, Satoru Murata, Ryoya Sakakibara, Takuma Aoki, Jun Koizumi

**Affiliations:** 1 Department of Cardiovascular Surgery, National Hospital Organization Saitama Hospital, Wako, JPN; 2 Department of Gastroenterology and Hepatology, National Hospital Organization Saitama Hospital, Wako, JPN; 3 Department of Radiology, National Hospital Organization Saitama Hospital, Wako, JPN

**Keywords:** acute cholangitis, celiac axis stenosis, duodenal obstruction, endoscopic nasobiliary drainage, median arcuate ligament syndrome, pancreaticoduodenal artery aneurysm, retroperitoneal hematoma, transcatheter arterial embolization, type b aortic dissection, visceral artery aneurysm

## Abstract

Aneurysms of the pancreaticoduodenal arcade are uncommon visceral artery aneurysms that are strongly associated with stenosis or occlusion of the celiac axis and that may rupture irrespective of size. Rupture produces a peripancreatic or retroperitoneal hematoma, and duodenal stenosis is a recognized sequela; biliary complications have rarely been reported. An 84-year-old man admitted with an acute Stanford type B aortic dissection with a thrombosed false lumen was managed conservatively with strict blood pressure control. Surveillance non-contrast computed tomography (CT) on day 7 incidentally showed a periduodenal hematoma, absent on day 5, in a patient who had no abdominal pain. Contrast-enhanced CT on day 8 demonstrated a 5-mm aneurysm of the anterior inferior pancreaticoduodenal artery (AIPDA) adjacent to the hematoma, together with severe celiac origin stenosis. The same narrowing was present on the day 0 study, as demonstrated on retrospective review. Coil embolization on day 12 occluded the aneurysm, and the arcade remained patent, maintaining collateral blood flow to the liver; the celiac origin stenosis was not treated. Postprandial vomiting began on day 16, and imaging on day 19 confirmed high-grade stenosis at the distal descending duodenum. He was managed with nasogastric decompression and parenteral nutrition, and endoscopy showed a short, tight stenosis of extrinsic appearance with normal mucosa. On day 37, he developed septic shock with direct-predominant hyperbilirubinemia, and CT showed a dilated biliary tree with severe narrowing of the common bile duct at the level of the coils. According to the Tokyo Guidelines 2018, grade III acute cholangitis was diagnosed, and an endoscopic nasobiliary drainage catheter was placed without sphincterotomy; blood and bile grew extended-spectrum beta-lactamase-producing *Escherichia coli*. The duodenal stenosis improved sufficiently for an oral elemental diet on day 45 and a full oral diet on day 47, and he was discharged home walking unaided on day 60. Rupture of an AIPDA aneurysm may be painless, although this is not the usual presentation, and may be detected only on surveillance imaging; severe cholangitis may follow in a patient with a persistent periduodenal hematoma and duodenal obstruction. Both complications were managed nonoperatively.

## Introduction

The pancreaticoduodenal arcade is the principal collateral pathway between the celiac and superior mesenteric arterial territories, running within and behind the head of the pancreas in contact with the duodenal wall. Aneurysms of the arcade account for an estimated 2% to 10% of visceral artery aneurysms but are clinically important because their risk of rupture is independent of diameter: rupture has been documented repeatedly in lesions smaller than 20 mm, and contemporary series recommend treatment at diagnosis regardless of size [1,2]. In a systematic review of 113 inferior pancreaticoduodenal artery aneurysms, 70.2% had already ruptured at presentation, and overall mortality was 9.6% [3].

These aneurysms are strongly associated with stenosis or occlusion of the celiac axis, most often from compression by the median arcuate ligament. When celiac inflow is restricted, the arcade hypertrophies as a collateral pathway and is exposed to chronically increased flow and wall stress [4,5]. Celiac origin stenosis was identified in 71.7% of the cases in that review [3].

Because the arcade lies in contact with the duodenal wall, rupture results in a peripancreatic or retroperitoneal hematoma rather than a free intraperitoneal hemorrhage. Duodenal stenosis is a recognized consequence, described both as the presenting feature and as a delayed event after transcatheter arterial embolization (TAE), and the reported cases differ in whether conservative management sufficed [6-9]. Biliary complications are far less often described, and two mechanisms have been reported: mass effect from the aneurysm or the associated hematoma, the mechanism in the cases collected in a review of obstructive jaundice complicating these aneurysms [10]; and direct rupture into the common bile duct, producing hemobilia and fatal acute obstructive suppurative cholangitis [11].

We report an elderly man in whom rupture of an anterior inferior pancreaticoduodenal artery (AIPDA) aneurysm was detected incidentally, in the absence of any abdominal pain, during conservative management of an acute type B aortic dissection and was followed by high-grade duodenal obstruction and then grade III acute cholangitis with bacteremia. Each of these events has been reported previously; we wish to document the sequence in a single patient. We document this sequence with serial imaging and endoscopic correlation and describe the rationale for nonoperative management.

## Case presentation

Presentation and initial management

An 84-year-old man was brought to our hospital by ambulance with sudden chest and back pain that had begun approximately one hour earlier. The day of arrival is designated day 0.

His history included hypertension treated with losartan, esaxerenone, and amlodipine; a prolactinoma resected in 1996 without recurrence; benign prostatic hyperplasia; and insomnia. He was taking neither antiplatelet nor anticoagulant therapy.

He was alert on arrival. Blood pressures were 197/126, 179/129, 198/122, and 204/126 mmHg in the right arm, left arm, right leg, and left leg, respectively, and the electrocardiogram showed sinus rhythm. Admission laboratory data are given in Table 1; the platelet count was mildly reduced, and D-dimer was elevated, while hepatobiliary enzymes and bilirubin were normal. Contrast-enhanced computed tomography (CT) showed an acute Stanford type B aortic dissection with a thrombosed false lumen extending from the aortic arch to just proximal to the celiac origin, sparing the ascending aorta; the dissection did not extend into the celiac artery (Figure 1).

**Table 1:** Laboratory findings Day 0 is the day of arrival with acute type B aortic dissection, and day 77 is the first outpatient visit, 17 days after discharge. Dashes indicate tests not performed on that day. Day 28 is shown because no sample was taken on day 29, the day of the first endoscopy. ALP was measured by the IFCC method. eGFR was estimated with the Japanese Society of Nephrology equation for Japanese adults: eGFR (mL/min/1.73 m²) = 194 x serum creatinine^-1.094 x age^-0.287 (x 0.739 if female). ALP: alkaline phosphatase, ALT: alanine aminotransferase, AST: aspartate aminotransferase, eGFR: estimated glomerular filtration rate, IFCC: International Federation of Clinical Chemistry and Laboratory Medicine, PT-INR: prothrombin time-international normalized ratio

Parameter (unit)	Reference range	Day 0	Day 8	Day 19	Day 28	Day 36	Day 37	Day 38	Day 39	Day 40	Day 48	Day 77
White blood cells (x10³/µL)	3.3-8.6	6.3	7.5	8.4	5.8	5.1	8.8	7.0	6.5	3.7	3.9	5.1
Neutrophils (%)	42-74	72.6	-	-	73.3	76.1	96.5	97.1	92.1	81.2	70.8	-
Hemoglobin (g/dL)	13.7-16.8	14.3	11.5	12.6	11.1	10.7	10.8	9.8	10.8	10.2	10.5	11.5
Platelets (x10³/µL)	158-348	119	146	285	104	100	91	75	81	76	149	133
PT-INR	0.90-1.13	0.92	1.04	-	-	-	1.05	1.35	1.28	1.04	1.04	-
D-dimer (µg/mL)	≤1.0	1.5	10.2	-	-	-	-	9.7	7.0	6.0	3.7	-
Total protein (g/dL)	6.6-8.1	6.7	-	-	-	-	-	4.8	-	-	-	6.3
Albumin (g/dL)	4.1-5.1	4.1	3.2	3.5	3.1	3.1	3.1	-	2.6	2.6	3.1	3.8
Total bilirubin (mg/dL)	0.4-1.5	0.7	0.9	1.1	1.4	1.1	2.9	3.2	2.6	1.8	1.1	0.9
Direct bilirubin (mg/dL)	0.0-0.4	0.2	-	-	-	-	2.4	2.7	1.8	1.2	0.6	0.3
AST (U/L)	13-30	15	17	15	19	19	168	101	43	30	19	14
ALT (U/L)	10-42	21	19	16	20	22	198	168	117	79	28	13
ALP, IFCC (U/L)	38-113	-	78	79	86	91	-	238	239	192	117	94
γ-Glutamyl transferase (U/L)	13-64	18	19	20	38	31	252	184	155	122	64	22
Amylase (U/L)	44-132	66	38	45	33	33	28	23	21	19	39	58
Lipase (U/L)	13-55	-	-	-	-	-	16	-	-	-	-	-
Blood urea nitrogen (mg/dL)	8.0-20.0	20.1	15.5	26.5	16.7	15.3	27.2	38.7	34.2	20.3	12.7	17.3
Creatinine (mg/dL)	0.65-1.07	1.01	0.96	1.16	0.96	0.90	1.41	1.88	1.19	0.93	0.76	0.91
eGFR (mL/min/1.73 m²)	-	53.8	56.9	46.2	56.9	61.0	37.3	27.3	45.0	58.9	73.4	60.3
C-reactive protein (mg/dL)	0.00-0.14	0.42	7.24	4.89	1.65	0.52	2.59	10.72	12.19	7.62	0.34	0.20

**Figure 1:**
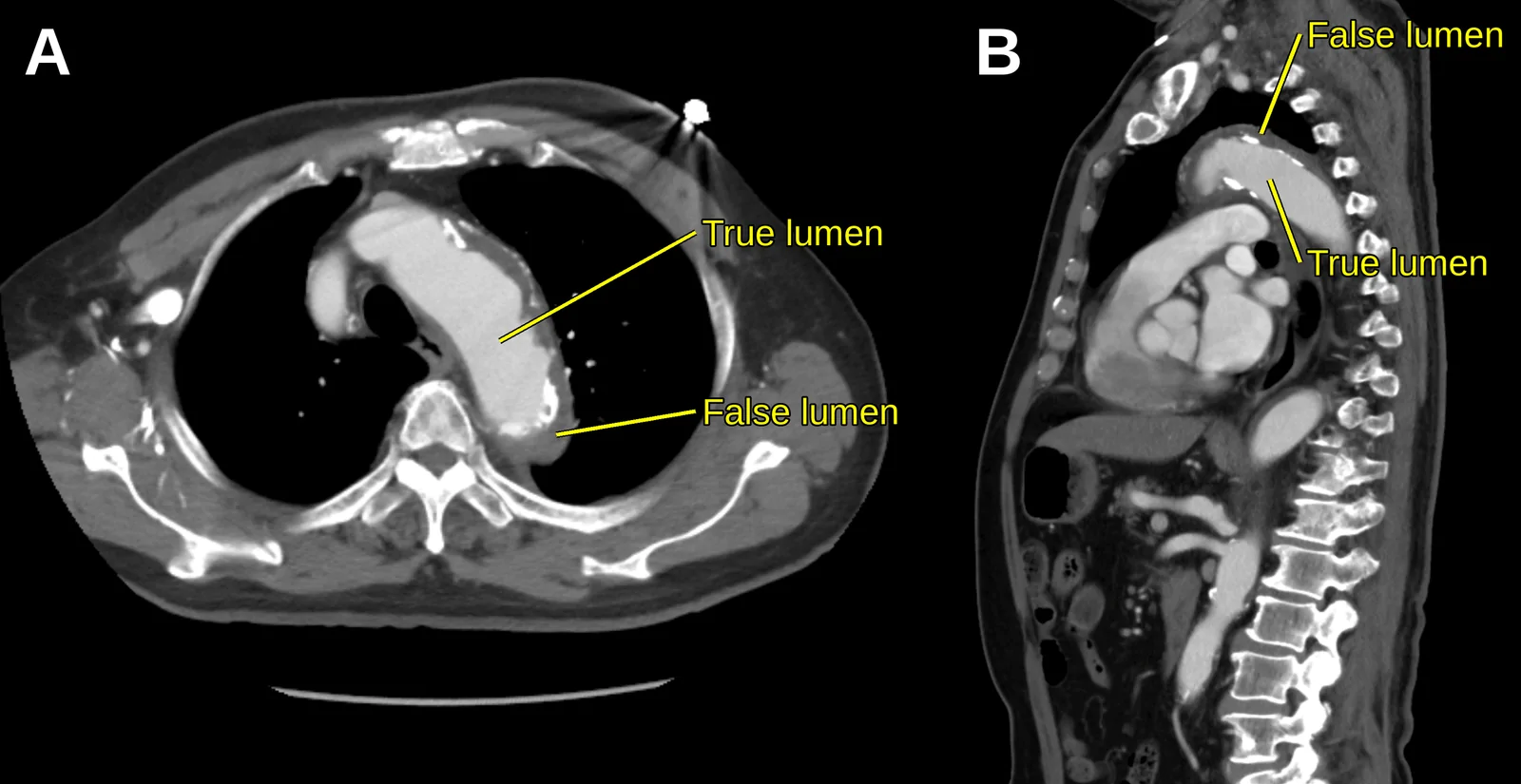
Contrast-enhanced CT on day 0 Axial (A) and sagittal (B) images show an acute Stanford type B aortic dissection with a thrombosed false lumen; the true and the false lumen are labeled on both panels. The dissection extended from the aortic arch to a level just proximal to the origin of the celiac artery. The ascending aorta was not involved, and the dissection did not extend into the celiac artery or its origin. CT: computed tomography

He was admitted to the intensive care unit for bed rest, analgesia and antihypertensive therapy as recommended for uncomplicated type B dissection [12], and reported no further pain. Intravenous nicardipine and nitroglycerin were used initially, with a target systolic pressure below 120 mmHg; oral agents (nifedipine, azilsartan and bisoprolol) were added from day 1, and the intravenous agents stopped on day 2. Thereafter, the systolic pressure remained mostly between 100 and 120 mmHg, not exceeding 140 mmHg until day 7. Oral intake, withheld on day 0, was resumed on day 1 and well tolerated.

Coronary CT angiography on day 2, followed immediately by a venous-phase acquisition of the chest, abdomen and pelvis, showed normal coronary arteries, with the false lumen still thrombosed and no aortic enlargement; non-contrast CT on day 5 was unchanged. He left the intensive care unit on day 5, was mobilizing well, and was expected to be discharged home.

Incidental detection and treatment of the ruptured AIPDA aneurysm

Surveillance non-contrast CT on day 7 showed a soft-tissue density consistent with hematoma around the descending and horizontal portions of the duodenum (Figure 2B). This finding was absent from the examinations on days 2 and 5 (the day 5 study is shown in Figure 2A). The patient had no abdominal pain, and the finding was entirely incidental. No oral or intravenous analgesic was administered between the CT examinations on days 5 and 7, and the daily nursing record documented no pain during that interval, so the absence of symptoms was not masked by analgesia.

**Figure 2:**
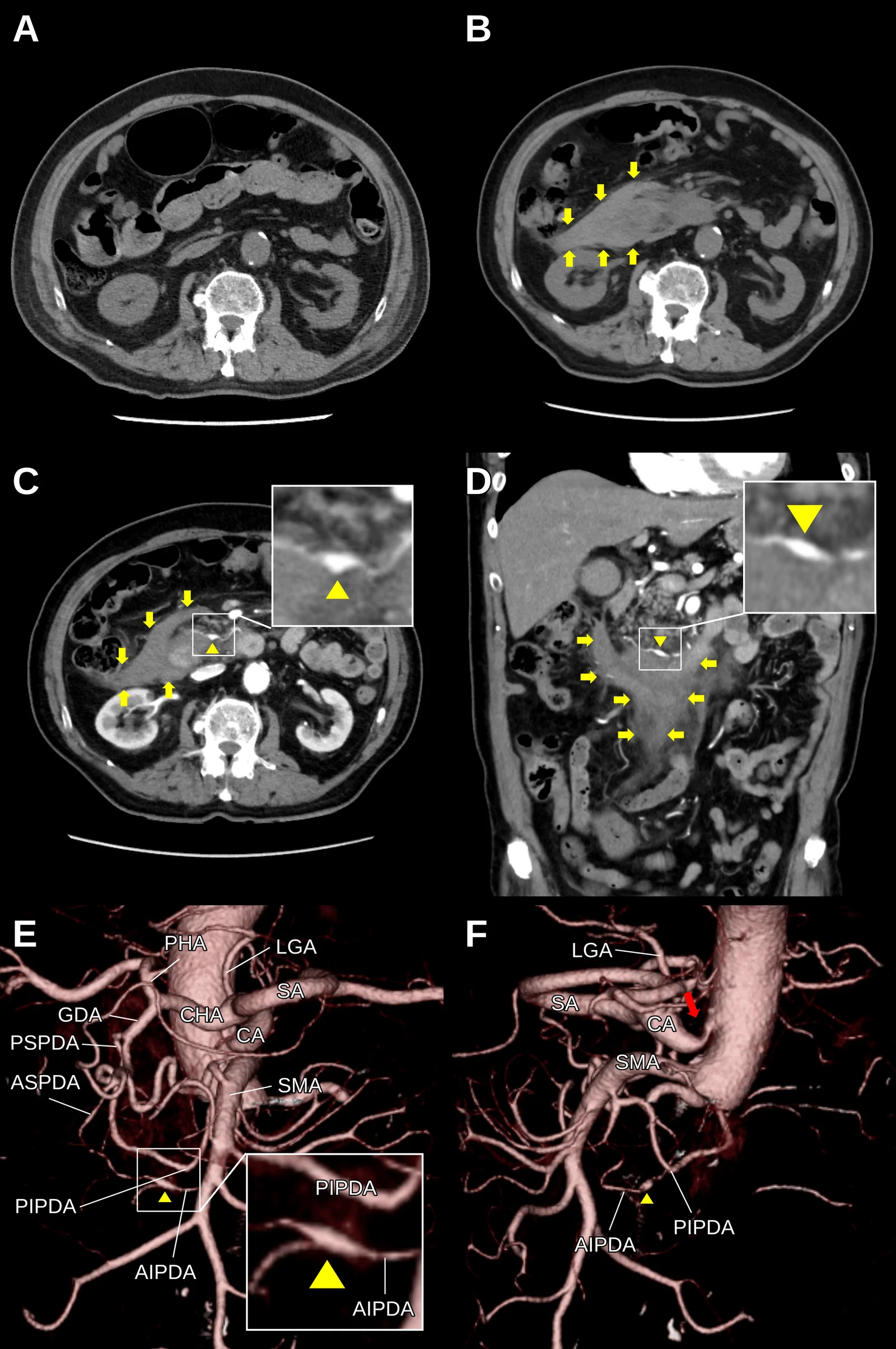
Detection of the ruptured AIPDA aneurysm (A-C) Axial CT images at an identical level, the upper third of the second lumbar vertebral body; the aortic calcifications at the 1 and 7 o'clock positions are depicted identically on all three panels and confirm the level. (A) Non-contrast CT on day 5; no periduodenal hematoma is present. (B) Non-contrast CT on day 7 showing a newly appeared periduodenal hematoma (yellow arrows). (C, D) Contrast-enhanced CT on day 8, axial (C) and coronal (D), showing the hematoma (yellow arrows) and the adjacent 5-mm AIPDA aneurysm (yellow arrowhead). (E, F) Three-dimensional volume-rendered reconstructions from the day 8 contrast-enhanced CT, with the vessels labeled on the images, showing the aneurysm (yellow arrowhead) arising from the AIPDA: frontal view (E), and left anterior oblique view (F), which also shows severe stenosis at the origin of the celiac artery (red arrow) with an appearance suggestive of median arcuate ligament compression. In (C), (D), and (E), the white box marks the region magnified in the inset, in which the arrowhead indicates the same aneurysm. AIPDA: anterior inferior pancreaticoduodenal artery, ASPDA: anterior superior pancreaticoduodenal artery, CA: celiac artery, CHA: common hepatic artery, CT: computed tomography, GDA: gastroduodenal artery, LGA: left gastric artery, PHA: proper hepatic artery, PIPDA: posterior inferior pancreaticoduodenal artery, PSPDA: posterior superior pancreaticoduodenal artery, SA: splenic artery, SMA: superior mesenteric artery

Contrast-enhanced CT on day 8 showed that the collection, which extended into the retroperitoneum, was unchanged and without contrast extravasation. Adjacent to it was a 5-mm aneurysm of the AIPDA, and the collection was interpreted as a contained rupture (Figure 2C-2D). Three-dimensional reconstruction demonstrated the aneurysm (Figure 2E-2F) and severe stenosis at the celiac origin, with an appearance suggestive of median arcuate ligament compression (Figure 2F); calcification of the celiac artery was minimal, and no atheromatous plaque was identified at the site of stenosis. Retrospective review of the day 0 study showed the same celiac narrowing, not appreciated prospectively, and no finding corresponding to the AIPDA aneurysm; the AIPDA itself was only just discernible because of its small caliber. The dissection did not involve the celiac artery on any of the studies obtained on days 0, 2, and 8. Oral intake was withheld; he remained afebrile, and two sets of blood cultures were negative.

Because there was no contrast extravasation and the vital signs were stable, embolization was scheduled electively and carried out on day 12. Angiography through a right common femoral approach, with the catheter in the inferior pancreaticoduodenal artery (IPDA), showed the AIPDA to be attenuated along its entire course from its origin, with an irregular fusiform aneurysm at its mid-portion; the posterior inferior pancreaticoduodenal artery (PIPDA), by contrast, was relatively dilated. There was no extravasation of contrast medium (Figure 3A). A microcatheter was advanced beyond the aneurysm and the sac was packed with one 3 mm × 6 cm detachable coil and three 2 mm × 6 cm pushable coils, occluding it. A further coil intended for the proximal neck became lodged in the microcatheter and was withdrawn with it; no coil remained outside the sac. Injection into the IPDA then no longer opacified the aneurysm and showed contrast passing through the PIPDA and the posterior superior pancreaticoduodenal artery (PSPDA) to the gastroduodenal and hepatic arteries, indicating that the arcade remained patent as a collateral pathway to the liver (Figure 3B). No treatment was directed at the celiac origin stenosis. Water and diet were resumed on days 13 and 14, and CT on day 14 confirmed a satisfactory result without active bleeding.

**Figure 3:**
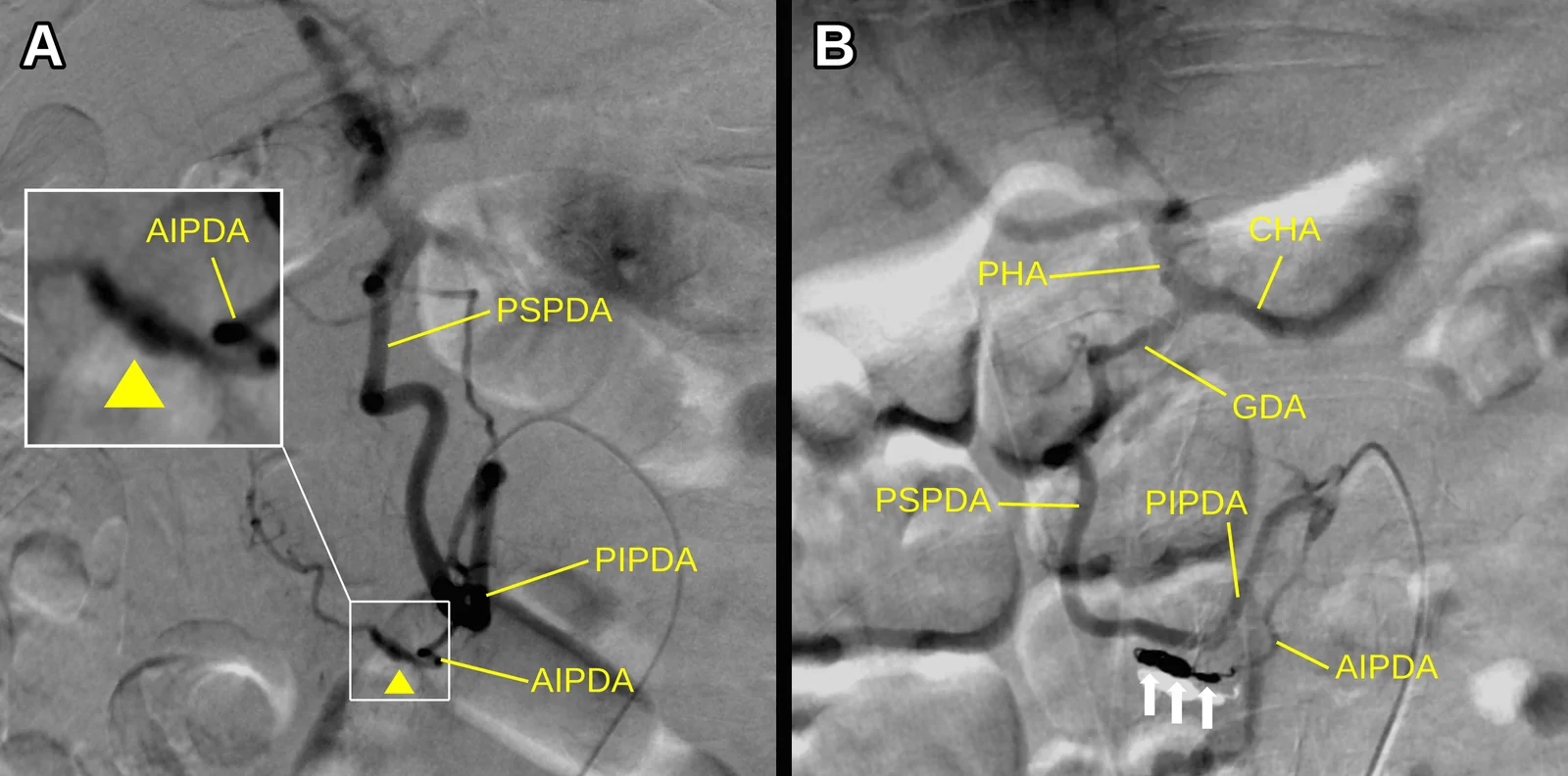
Digital subtraction angiography before and after coil embolization on day 12 Both panels were obtained with the catheter positioned in the IPDA, and the vessels are labeled on the images. (A) Before embolization. An irregular fusiform aneurysm (yellow arrowhead) arises from the mid-portion of the AIPDA and is shown magnified in the inset marked by the white box; the PIPDA and the PSPDA are also opacified. There is no extravasation of contrast medium. (B) After embolization, the coils packed into the sac are indicated (white arrows). The aneurysm is no longer opacified, and contrast passes from the IPDA through the PIPDA and PSPDA to the GDA, CHA, and PHA, demonstrating that the pancreaticoduodenal arcade remains patent as a collateral pathway to the liver. AIPDA: anterior inferior pancreaticoduodenal artery, CHA: common hepatic artery, GDA: gastroduodenal artery, IPDA: inferior pancreaticoduodenal artery, PHA: proper hepatic artery, PIPDA: posterior inferior pancreaticoduodenal artery, PSPDA: posterior superior pancreaticoduodenal artery

Duodenal obstruction

Vomiting occurred after meals on days 16 and 17. Non-contrast CT on day 18 showed a distended, fluid-filled stomach with the duodenum collapsed distal to the level of the coils, and contrast-enhanced CT on day 19 confirmed high-grade stenosis at the distal descending duodenum with fluid-filled distension extending proximally to the esophagus (Figure 4). A nasogastric tube placed the same day drained 1800 mL immediately, and output remained 300-1100 mL per day until day 31, then fell to about 200 mL per day. A peripherally inserted central catheter was placed on day 20, and total parenteral nutrition was started.

**Figure 4:**
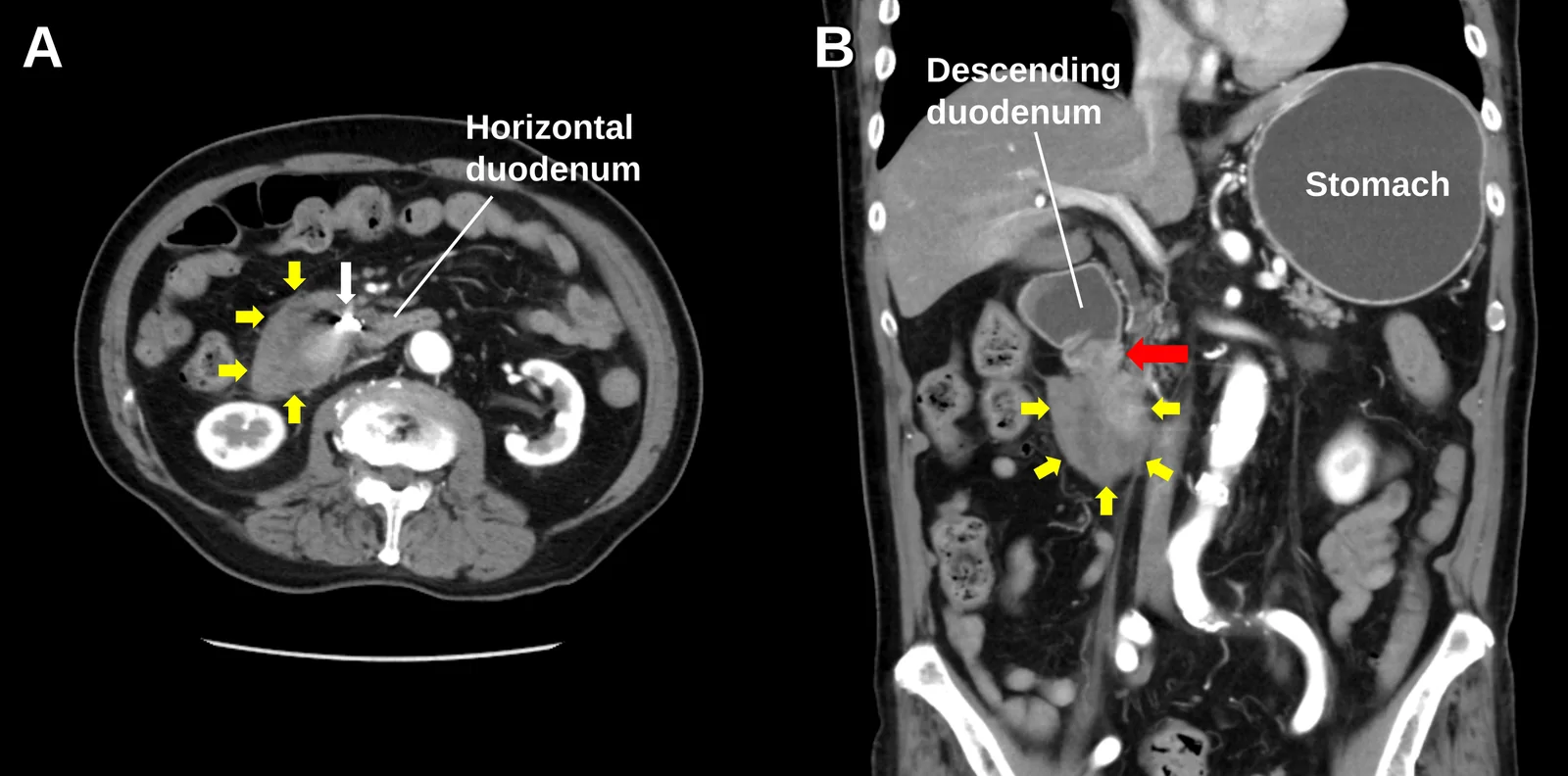
Contrast-enhanced CT on day 19 In both panels, the yellow arrows indicate the periduodenal hematoma and the white arrow the coils used to embolize the AIPDA. (A) Axial image; the horizontal duodenum, distal to the stenosis, is not dilated. (B) Coronal image; the red arrow indicates the duodenal stenosis, and the stomach and the duodenum proximal to the stenosis are markedly dilated and filled with fluid. AIPDA: anterior inferior pancreaticoduodenal artery

Esophagogastroduodenoscopy on day 29 showed a localized stenosis of the distal descending duodenum that could not be traversed with a standard-caliber gastroscope (outer diameter 9.9 mm); the mucosa overlying the stenosis was normal, without tumor or ulceration (Figure 5A). The examination was repeated with an ultrathin transnasal gastroscope (distal-end diameter 5.4 mm), which passed through the stenosis and showed that the narrowing was confined to a short segment, with normal mucosa immediately beyond it. The appearances were attributed to extrinsic compression by the hematoma with an accompanying inflammatory reaction.

**Figure 5:**
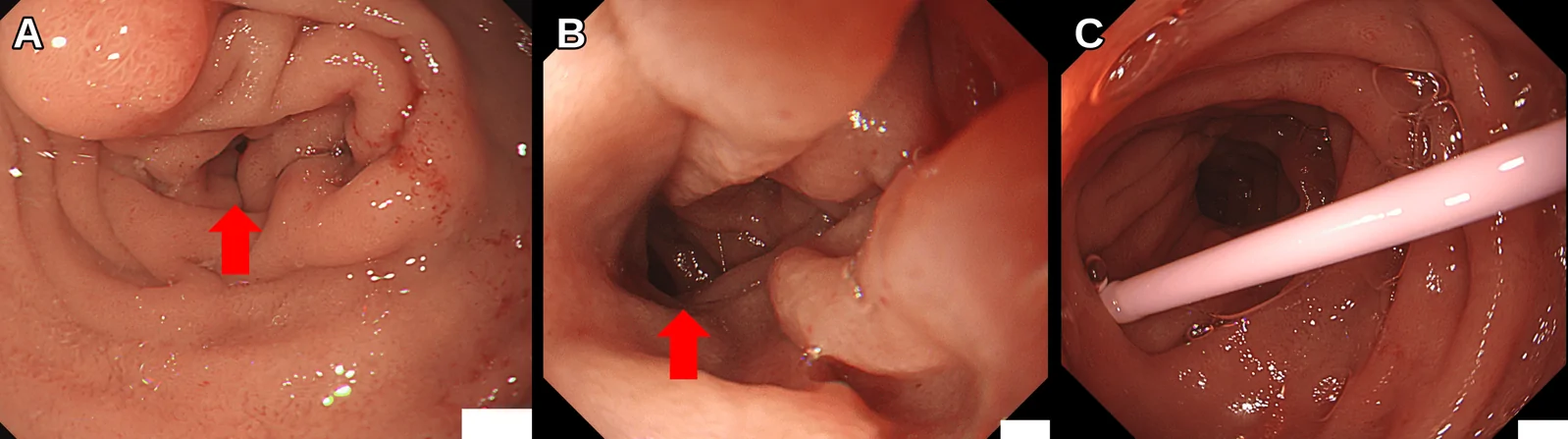
Serial esophagogastroduodenoscopy of the distal descending duodenum In (A) and (B), the red arrow indicates the duodenal stenosis. (A) Day 29: a tight stenosis that could not be traversed with the standard-caliber gastroscope (outer diameter 9.9 mm); the overlying mucosa is normal, and the punctate reddening is contact change from the endoscope, without erosion, ulceration, or necrosis. An ultrathin transnasal gastroscope (distal-end diameter 5.4 mm) was passed through the stenosis at this examination (not shown). (B) Day 36: the stenosis distends slightly with insufflation, and the mucosa remains normal. (C) Day 44: the stenosis has improved and admits the standard-caliber gastroscope. The tubular structure crossing the field is the ENBD catheter. The white rectangle in the lower right corner of each panel masks the image timestamp. ENBD: endoscopic nasobiliary drainage

On day 35, the peripherally inserted central catheter was exchanged for a central venous catheter via the right internal jugular vein. Endoscopy on day 36, with the standard-caliber gastroscope alone, showed that the stenosis distended slightly with insufflation, that the mucosa remained normal, and that there was no progressive ischemic change (Figure 5B); the nasogastric tube was removed, and an oral elemental diet was begun.

Grade III acute cholangitis and biliary drainage

Before noon on day 37, the patient was found to have a supine blood pressure of 68/52 mmHg with fever and rigors. Initial circulatory management with noradrenaline and volume resuscitation was begun. At the same time, the laboratory results were awaited, and the oral elemental diet started the previous day was stopped and a nasogastric tube reinserted. Testing showed direct-predominant hyperbilirubinemia, marked elevations of transaminases and γ-glutamyl transferase, and a rising C-reactive protein.

Contrast-enhanced CT showed a dilated common bile duct, cystic duct, and gallbladder without an impacted calculus, and severe narrowing of the common bile duct at the same level as the embolization coils and the periduodenal inflammatory change (Figure 6A). The diagnosis satisfied all three Tokyo Guidelines 2018 (TG18) domains for acute cholangitis, and the severity was grade III [13]. Although the platelet count was below 100 x 10³/µL, thrombocytopenia had preceded this episode; cardiovascular dysfunction requiring vasopressor support by itself satisfied the grade III criteria.

**Figure 6:**
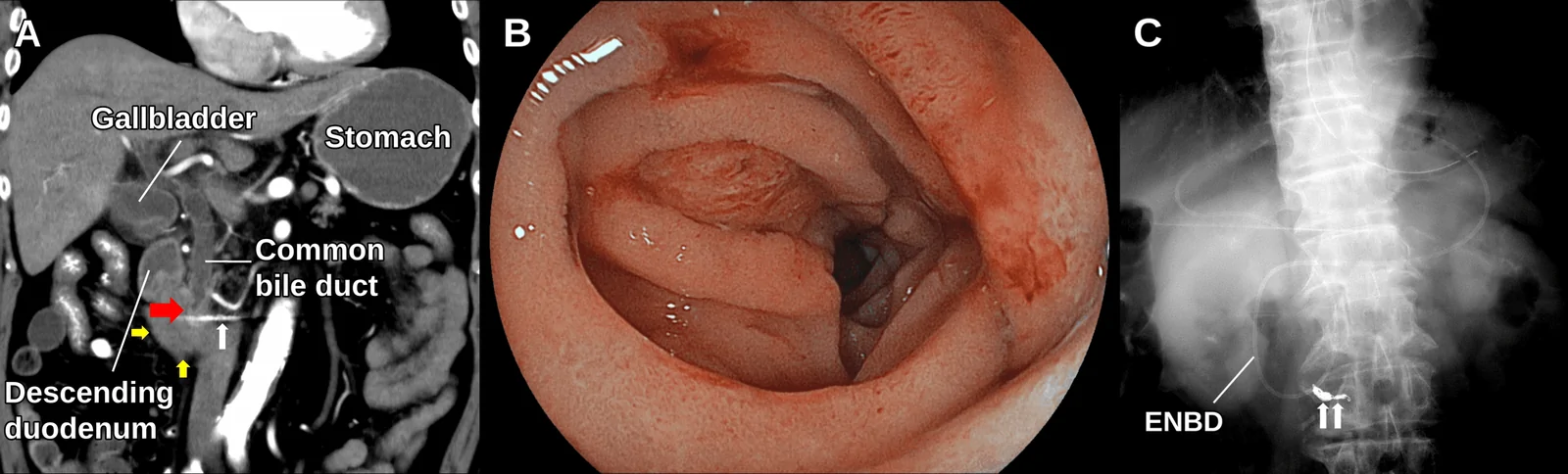
Grade III acute cholangitis on day 37 In (A), the yellow arrows indicate the periduodenal hematoma, the white arrow the artifact from the coils used to embolize the AIPDA, and the red arrow the site of narrowing of the common bile duct. (A) Coronal contrast-enhanced CT. The common bile duct is dilated without an impacted calculus and is severely narrowed at the same level as the coils; the stomach, gallbladder, common bile duct, and descending duodenum are labeled. (B) Endoscopic view showing marked edema of the duodenal mucosa surrounding the major papilla. (C) Plain abdominal radiograph obtained at the end of the procedure, displayed at a window chosen to make the nasobiliary catheter visible; the white arrows indicate the coils, which appear to lie at the same level as the major papilla. AIPDA: anterior inferior pancreaticoduodenal artery, CT: computed tomography, ENBD: endoscopic nasobiliary drainage

Two sets of blood cultures were taken, and empirical intravenous meropenem was started the same day; both sets grew extended-spectrum beta-lactamase (ESBL)-producing *Escherichia coli*, confirming that the empirical regimen was appropriate [14,15]. Emergency therapeutic endoscopic retrograde cholangiopancreatography (ERCP) was undertaken the same evening. Edema of the descending duodenum made an en face view of the major papilla impossible with the duodenoscope, and the papilla became visible only after switching to a slim therapeutic gastroscope. After guidewire cannulation, a 5-Fr endoscopic nasobiliary drainage (ENBD) catheter was placed without sphincterotomy and without contrast cholangiography (Figure 6B-6C); no cholangiographic images are therefore available. Bile obtained via the catheter on day 38 yielded the same organism, confirming a biliary source. The central venous catheter was removed on day 39 due to bacteremia and was not replaced; thereafter, parenteral nutrition was administered via a peripheral route.

Recovery and outcome

By day 40, he had improved; hepatobiliary enzymes and inflammatory markers were falling, and the coagulation abnormalities were resolving (Table 1). Noradrenaline was stopped, and repeat blood cultures were negative. Endoscopy on day 44 showed that the stenosis now allowed passage of a standard-caliber gastroscope, with no erosion or ulceration (Figure 5C). The ENBD catheter was removed. An oral elemental diet was restarted on day 45 with the nasogastric tube left in place, and the tube was removed on day 47, when a full oral diet was resumed. Meropenem was completed on day 48 and peripheral parenteral nutrition on day 50. CT on day 51 showed no dilatation of the stomach, duodenum, or common bile duct, a marked reduction in the periduodenal hematoma, and no new aneurysm; the false lumen remained thrombosed and had decreased in size. He was discharged home on day 60, walking unaided and independent in daily activities, although his weight had fallen from 71.5 kg to 58.9 kg. Oral nutrition had been withheld for most of the interval between day 19 and the elemental diet of day 45. It was replaced with parenteral nutrition on the advice of the nutrition support team, and serum albumin had reached a nadir of 2.6 g/dL (Table 1). No enteral feeding tube was placed beyond the stenosis, and neither duodenal stenting nor gastrojejunostomy was performed. The course is summarized in Figure 7.

**Figure 7:**
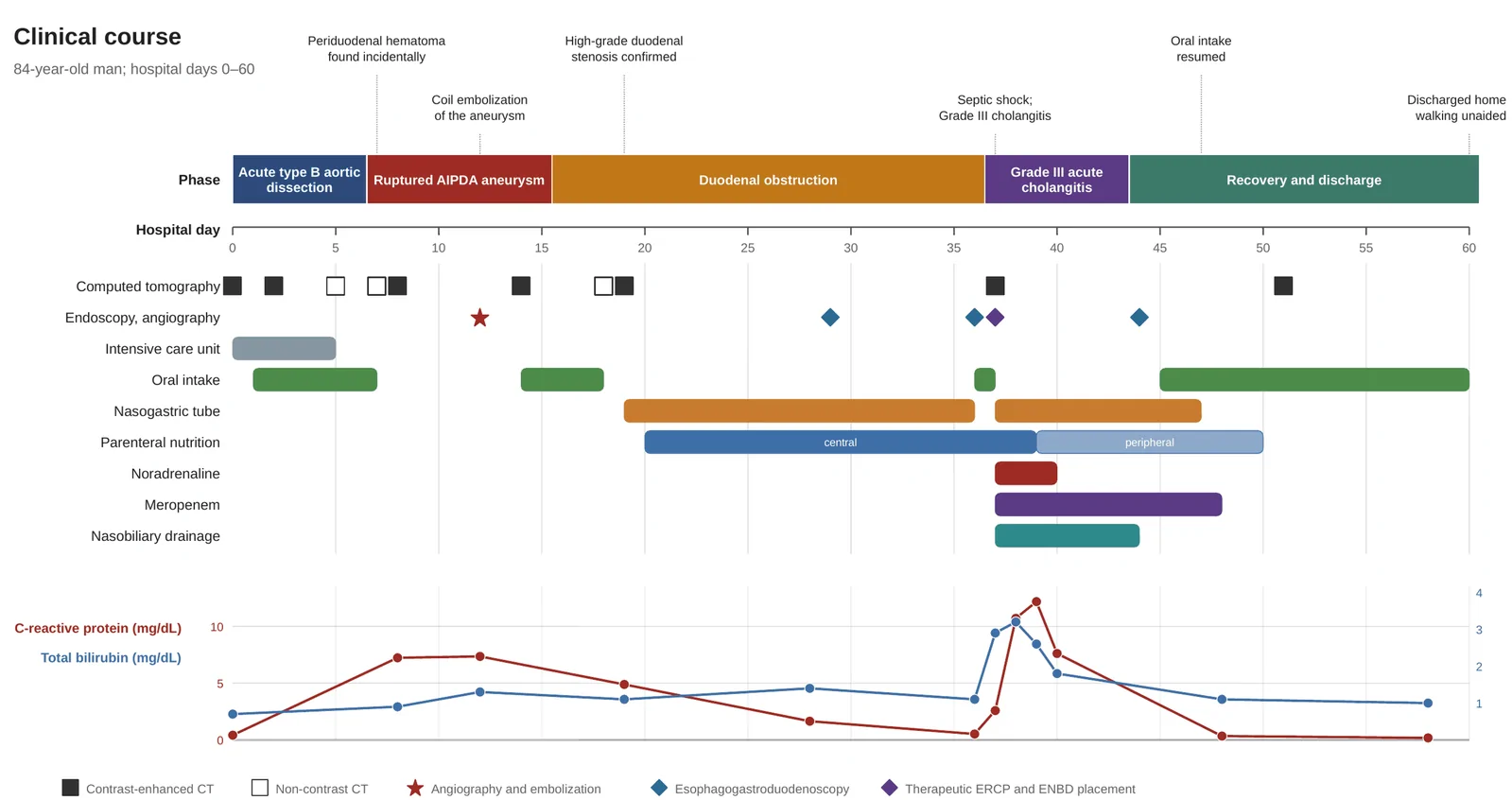
Graphical timeline of the clinical course The five phases of the admission are shown as colored bands above a scale of hospital days. In the examination rows, the symbols denote: filled square, contrast-enhanced CT; open square, non-contrast CT; star, angiography with coil embolization of the AIPDA aneurysm; teal diamond, esophagogastroduodenoscopy; purple diamond, therapeutic ERCP with placement of an ENBD catheter. Horizontal bars indicate the periods of intensive care, oral intake, nasogastric decompression, parenteral nutrition, noradrenaline, meropenem, and nasobiliary drainage; the single narrow bar on day 36 denotes the oral elemental diet that was begun at the second endoscopy and interrupted the next day by the onset of cholangitis. The parenteral nutrition bar is divided at day 39, where the route changed from central to peripheral. The lower panel shows C-reactive protein (left axis) and total bilirubin (right axis) over the course of admission. AIPDA: anterior inferior pancreaticoduodenal artery, CT: computed tomography, ENBD: endoscopic nasobiliary drainage, ERCP: endoscopic retrograde cholangiopancreatography

When seen in the clinic 17 days after discharge (day 77), he was well, eating normally and afebrile, with normal liver enzymes and with albumin and total protein rising (Table 1). Given his age, the plan is medical management centered on blood pressure control, with surveillance CT within the next few months and at intervals thereafter, and intervention if a new or enlarging aneurysm is demonstrated or if symptoms develop.

## Discussion

Three processes that are individually well described occurred here in sequence: rupture of a pancreaticoduodenal arcade aneurysm, duodenal obstruction from the resulting hematoma, and severe secondary cholangitis. Set against the review of 113 inferior pancreaticoduodenal artery aneurysms, the lesion itself was typical, but the clinical course was not: rupture before detection and celiac origin stenosis placed our patient within both major groups described in that review [3], but neither the collected cases of duodenal stenosis after embolization nor the reported biliary complications of these aneurysms combine all three of the events seen here [8,10,11].

An acute lesion on a chronically remodeled arcade

Severe stenosis of the celiac origin, with features suggestive of median arcuate ligament compression, was present on day 8 (Figure 2F) and, in retrospect, on the day 0 study; the minimal calcification and the absence of plaque favor extrinsic compression over the atherosclerotic stenosis that would otherwise be expected at this age. The precondition for the classic pathophysiology of these aneurysms, restricted celiac inflow with the arcade carrying flow redistributed from the superior mesenteric artery, was therefore present [4,5]. At angiography, the posterior arcade appeared dominant: the PIPDA was relatively dilated, consistent with chronic collateral flow, whereas the AIPDA, which bore the aneurysm, was attenuated along its whole length rather than focally and was already of small caliber on the day 0 study, so the adjacent hematoma is unlikely to explain the attenuation. The anterior channel therefore appears to have been the smaller of the two before the rupture; why the aneurysm arose there rather than on the dilated posterior channel cannot be determined from a single case. The aneurysm measured only 5 mm, consistent with the observation that rupture of these lesions is not predicted by size [1,2].

What we cannot explain is the timing. No hematoma was present on day 5, and no finding corresponding to the aneurysm could be identified on the day 0 study. Two readings are possible: a small true aneurysm may have been present throughout but not resolved on the earlier study, because a 5-mm lesion on a peripheral arcade branch is at the limit of what such an examination can demonstrate; alternatively, the lesion may have been a pseudoaneurysm created by the rupture itself. Imaging alone cannot distinguish them, although the causes that ordinarily produce a pseudoaneurysm here (pancreatitis, blunt trauma, and preceding instrumentation) were all absent, and the arcade was exposed to the celiac stenosis under which true aneurysms characteristically develop. Nor can we identify a precipitant. From day 2 onward, systolic pressure never exceeded 140 mmHg until day 7, so initial hypertension cannot reasonably be invoked. The dissection stopped just above the celiac origin, did not extend into the celiac artery in any study, and had a thrombosed false lumen without compression of the true lumen. Hence, an acute change in visceral arterial inflow is difficult to postulate. The relationship between the dissection and the rupture therefore remains unclear.

Duodenal obstruction: mechanism and management

Duodenal stenosis after rupture of a pancreaticoduodenal artery aneurysm is reported both as the presenting feature and as a delayed event. Obstruction at presentation has been attributed to an intramural duodenal hematoma [6], to a 19-cm retroperitoneal hematoma that resolved over two weeks after coil embolization and nasogastric decompression [7], and to a pseudoaneurysm with a pancreatic fibroinflammatory mass in which a regular diet was resumed four weeks after embolization [16]. Stenosis emerging only after TAE, as in our patient, was reviewed by Nakayama et al. in 12 cases: the median interval to onset was 10 days, 10 of the 12 arose in the subacute phase, and every patient improved with conservative management [8]. In our patient, the interval was four days, which may indicate a larger contribution from mechanical compression by the hematoma; a single case cannot settle the point.

Three mechanisms may have contributed: extrinsic compression by the hematoma; an inflammatory reaction to extravasated retroperitoneal blood, which also explains duodenal obstruction complicating spontaneous retroperitoneal hematoma from other causes, an obstruction that characteristically resolves over three to five weeks of conservative treatment [17]; and ischemic injury after embolization. Nakayama et al. argued that ischemia is unlikely after coil embolization because coils preserve the distal arteries and because the duodenum retains a rich collateral supply; they instead proposed that edema develops as the hematoma organizes [8]. Tanikawa et al. reached a similar conclusion in a patient whose stenosis appeared 15 days after TAE, attributing it to edematous change rather than to mechanical compression; in their patient, the mucosa showed edema without erosion or ulceration, but the stenosis did not improve over 12 days of conservative treatment, and median arcuate ligament release with gastrojejunostomy was performed on the 35th hospital day, with endoscopic resolution at four months [9].

The same reasoning applies to our patient. Embolization was confined to a peripheral arcade segment, and the post-embolization angiogram showed that the arcade continued to supply the hepatic arteries (Figure 3B). Three endoscopic observations argue against a significant ischemic contribution: the mucosa overlying the stenosis was normal on days 29 and 36 apart from endoscope contact change, without the erosion or necrosis expected with significant mural ischemia; the 5.4-mm instrument showed the narrowing to be confined to a short segment, which fits extrinsic compression with adjacent inflammatory edema better than a longer ischemic stricture; and the mucosa immediately distal to it, inspected with the ultrathin instrument, was also normal. The stenosis then improved incrementally rather than progressing, and the stenosis admitted the 9.9-mm gastroscope on day 44. The difference from the case of Tanikawa et al. therefore lies in the outcome rather than the mechanism; their operation was undertaken in part because the patient wished for a faster recovery, whereas in our patient the stenosis improved sufficiently for oral intake to be resumed without surgery. A contribution from transient mural hypoperfusion cannot be formally excluded.

Management was deliberately conservative: nasogastric decompression relieved distension, parenteral nutrition supported the patient during the prolonged fast, and serial endoscopy objectively documented improvement. Because serial endoscopy on days 36 and 44 showed sufficient improvement to allow removal of the nasogastric tube and resumption of oral intake, no enteral tube feeding beyond the stenosis, duodenal stenting, or gastrojejunostomy was judged necessary. Endoscopic reassessment is valuable both for excluding mucosal disease and for following the stenosis.

Embolization in the presence of celiac stenosis, and the decision not to treat the celiac lesion

When the celiac origin is severely stenotic, the pancreaticoduodenal arcade provides collateral supply to the liver, stomach, and spleen, and occluding it carries risk. In this patient, the coils were packed within the sac, and the arcade itself was preserved (Figure 3B); the aminotransferases and biliary enzymes remained normal until the septic episode on day 37.

Whether the celiac stenosis itself should also be treated is unsettled. Some authors report that celiac revascularization may be unnecessary in asymptomatic patients whose aneurysm has been occluded and who can be followed with serial imaging [18]. In contrast, others advocate a staged approach in which the aneurysm is excluded, and the celiac axis is then revascularized [19]. Endovascular treatment of extrinsic celiac compression carries its own hazard: in a series of four patients, both of those treated by stenting developed stent-associated thrombosis [20]. In our patient, the aneurysm arose from the smaller of the two inferior arcade branches; the arcade remained patent after embolization, and he was 84 years old and convalescing from an acute aortic dissection; observation was therefore chosen. In a younger patient with confirmed median arcuate ligament compression, laparoscopic division would have been considered.

Secondary acute cholangitis

Grade III acute cholangitis on day 37 was the most serious event of the admission. The distal common bile duct traverses the pancreatic head. It enters the duodenum at the major papilla in the descending portion, precisely the region occupied by the hematoma, the embolized segment, and the inflammatory reaction. CT showed a dilated biliary tree without a calculus and severe narrowing of the duct at the level of the coils (Figure 6A), and endoscopy showed marked periampullary edema (Figure 6B). Together, these findings suggest that the obstruction lay in the distal common bile duct and periampullary region and support periduodenal inflammation with mucosal edema as the most plausible cause; prolonged fasting and biliary stasis may also have contributed.

This mechanism differs from the biliary complications previously described with these aneurysms: direct rupture into the biliary system with hemobilia and fatal suppurative cholangitis [11] and obstruction by the aneurysm or the associated hematoma in the 11 cases of obstructive jaundice reviewed by Jalili et al., in which mortality was 45.4% [10]. In our patient, there was no hemobilia and no clear evidence of direct compression by the aneurysm itself; the narrowing appeared at the level of the coils and of the periampullary inflammation, after the aneurysm had been excluded and while the duodenum was obstructed. A contribution from mass effect by the adjacent hematoma cannot be excluded.

The elevation of the transaminases on day 37 warrants comment because three conditions favoring reduced hepatic perfusion coexisted: severe celiac origin stenosis, embolization within its principal collateral pathway, and septic shock requiring vasopressor support. Three findings, nonetheless, indicate that biliary obstruction and infection predominated: the hyperbilirubinemia was direct-predominant, CT showed a dilated biliary tree with narrowing of the distal duct, and bile from the catheter grew the same ESBL-producing *Escherichia coli* as the blood. A contribution from transient hepatic hypoperfusion during the shock episode cannot be excluded.

Management followed TG18 principles: prompt recognition, organ support, appropriate antimicrobial therapy, and urgent drainage [13,14]. Because this was a healthcare-associated infection after a prolonged admission, empirical carbapenem therapy was chosen at the outset, a decision supported by the subsequent culture results [15]. ENBD was preferred to stenting because it permits monitoring of drainage volume and character, allows repeated bile sampling, and can be removed without a further procedure. Patients with prolonged duodenal obstruction after arcade aneurysm rupture warrant active surveillance for biliary complications, and deterioration should not be attributed reflexively to the known duodenal problem.

Limitations

This is a single case, and the mechanistic explanations offered are inferential. The absence of pain should not be generalized: reported ruptures usually present with pain or hemodynamic instability, and this observation argues not for the expectation that such ruptures are commonly silent but for vigilance when imaging is obtained for another indication. Median arcuate ligament compression was not confirmed dynamically with expiratory imaging, so we have described the appearance rather than made the diagnosis. No tissue was obtained; therefore, an underlying arteriopathy, such as segmental arterial mediolysis, cannot be excluded. The imaging cannot establish when the lesion appeared: the day 2 abdominal study was a venous-phase acquisition, not suited to small visceral arteries, and the day 5 study was unenhanced. The biliary narrowing was characterized on CT alone because cholangiography was not performed. Finally, follow-up extends only 17 days beyond discharge.

## Conclusions

Rupture of an anterior inferior pancreaticoduodenal artery aneurysm may occur during the acute phase of a type B aortic dissection, may be entirely painless, and may be detected only on surveillance CT obtained for the aortic disease; whether the two events were causally related could not be determined. The resulting periduodenal hematoma produced high-grade duodenal obstruction, and severe acute cholangitis with bacteremia followed, in a distribution that suggests periampullary inflammation as the cause.

Both complications were managed nonoperatively. Nasogastric decompression, parenteral nutrition, and serial endoscopic reassessment enabled the duodenal stenosis to improve sufficiently for an oral elemental diet on day 45 and a full oral diet on day 47. Meanwhile, prompt recognition of grade III acute cholangitis, empirical carbapenem therapy, and ENBD controlled the septic episode. This is consistent with previous reports indicating that duodenal stenosis after embolization usually resolves with conservative treatment. New or recurrent vomiting after treatment of such an aneurysm should prompt early investigation for duodenal obstruction, and biliary sepsis should be considered as a delayed complication in patients with a persistent periduodenal hematoma and duodenal obstruction. Our patient was discharged home walking unaided after joint management by cardiovascular surgery, interventional radiology, and gastroenterology.

## References

[1] Stoecker JB, Eddinger KC, Glaser JD, Wang GJ, Shlansky-Goldberg RD, Fairman RM, Jackson BM (2022). A large series of true pancreaticoduodenal artery aneurysms. J Vasc Surg.

[2] Corey MR, Ergul EA, Cambria RP, Patel VI, Lancaster RT, Kwolek CJ, Conrad MF (2016). The presentation and management of aneurysms of the pancreaticoduodenal arcade. J Vasc Surg.

[3] Horowitz R, Hoffman S, Argaman M, Dadaev V, Shakkur W, Kanani F (2026). Inferior pancreaticoduodenal artery aneurysms: diagnostic challenges and management outcomes-a systematic review of 113 cases. Am Surg.

[4] Kalva SP, Athanasoulis CA, Greenfield AJ, Fan CM, Curvelo M, Waltman AC, Wicky S (2007). Inferior pancreaticoduodenal artery aneurysms in association with celiac axis stenosis or occlusion. Eur J Vasc Endovasc Surg.

[5] Murata S, Tajima H, Fukunaga T (2006). Management of pancreaticoduodenal artery aneurysms: results of superselective transcatheter embolization. AJR Am J Roentgenol.

[6] Reddy N, Metwalli ZA (2019). Ruptured pancreaticoduodenal artery aneurysm presenting with duodenal obstruction. Radiol Case Rep.

[7] Naing SS (2026). Pancreaticoduodenal artery aneurysm presenting with retroperitoneal haemorrhage and gastric outlet obstruction: a case report. J Surg Case Rep.

[8] Nakayama K, Shimohira M, Nagai K (2021). Duodenal stenosis after transcatheter arterial embolization for rupture of an inferior pancreaticoduodenal aneurysm. Radiol Case Rep.

[9] Tanikawa T, Miyake K, Kawada M (2025). Aneurysm rupture in median arcuate ligament syndrome leading to duodenal stenosis: a case report. World J Clin Cases.

[10] Jalili J, Javadrashid R, Alvandfar D, Falahatian M, Jafarizadeh A, Alihosseini S, Hashemizadeh SE (2023). Obstructive jaundice as a rare complication of multiple pancreaticoduodenal artery aneurysms due to median arcuate ligament syndrome: a case report and review of the literature. J Med Case Rep.

[11] Kurisu A, Matsuki M, Kawachi Y, Taki K (2005). Rupture of a pancreaticoduodenal artery aneurysm into the common bile duct resulting in fatal suppurative cholangitis: report of a case. Surg Today.

[12] Isselbacher EM, Preventza O, Hamilton Black J 3rd (2022). 2022 ACC/AHA guideline for the diagnosis and management of aortic disease: a report of the American Heart Association/American College of Cardiology joint committee on clinical practice guidelines. Circulation.

[13] Kiriyama S, Kozaka K, Takada T (2018). Tokyo Guidelines 2018: diagnostic criteria and severity grading of acute cholangitis (with videos). J Hepatobiliary Pancreat Sci.

[14] Miura F, Okamoto K, Takada T (2018). Tokyo Guidelines 2018: initial management of acute biliary infection and flowchart for acute cholangitis. J Hepatobiliary Pancreat Sci.

[15] Gomi H, Solomkin JS, Schlossberg D (2018). Tokyo Guidelines 2018: antimicrobial therapy for acute cholangitis and cholecystitis. J Hepatobiliary Pancreat Sci.

[16] Asrih M, Allali D, Morère PH, Guibert P, Kherad O (2024). Pancreaticoduodenal artery pseudoaneurysm associated with pancreatic fibroinflammatory mass and duodenal obstruction. Am J Case Rep.

[17] Makazu M, Koizumi K, Masuda S, Jinushi R, Shionoya K, Tsukiyama T (2023). Spontaneous retroperitoneal hematoma with duodenal obstruction with diagnostic use of endoscopic ultrasound: a case series and literature review. Clin J Gastroenterol.

[18] Sgroi MD, Kabutey NK, Krishnam M, Fujitani RM (2015). Pancreaticoduodenal artery aneurysms secondary to median arcuate ligament syndrome may not need celiac artery revascularization or ligament release. Ann Vasc Surg.

[19] Ghoneim B, Nash C, Akmenkalne L (2024). Staged treatment for pancreaticoduodenal artery aneurysm with coeliac artery revascularisation: case report and systematic review. Vascular.

[20] Takeuchi Y, Morikage N, Samura M (2017). Treatment options for celiac stenosis and pancreaticoduodenal artery aneurysms. Ann Vasc Surg.

